# Valveless On-Chip Aliquoting for Molecular Diagnosis

**DOI:** 10.3390/mi14071425

**Published:** 2023-07-15

**Authors:** Andersson A. Romero Deza, Federico Schaumburg, Claudio L. A. Berli

**Affiliations:** 1 Facultad de Ciencias, Universidad Nacional de Ingeniería, Lima 15333, Peru; 2 Predio CCT CONICET Santa Fe, INTEC (Universidad Nacional del Litoral-CONICET), RN 168, Santa Fe 3000, Argentina

**Keywords:** lab-on-a-chip, metering, molecular diagnosis, isothermal amplification, CRISPR/Cas

## Abstract

The detection of nucleic acids as specific markers of infectious diseases is commonly implemented in molecular biology laboratories. The translation of these benchtop assays to a lab-on-a-chip format demands huge efforts of integration and automation. The present work is motivated by a strong requirement often posed by molecular assays that combine isothermal amplification and CRISPR/Cas-based detection: after amplification, a 2–8 microliter aliquot of the reaction products must be taken for the subsequent reaction. In order to fulfill this technical problem, we have designed and prototyped a microfluidic device that is able to meter and aliquot in the required range during the stepped assay. The operation is achieved by integrating a porous material that retains the desired amount of liquid after removing the excess reaction products, an innovative solution that avoids valving and external actuation. The prototypes were calibrated and experimentally tested to demonstrate the overall performance (general fluidics, metering, aliquoting, mixing and reaction). The proposed aliquoting method is fully compatible with additional functions, such as sample concentration or reagent storage, and could be further employed in alternative applications beyond molecular diagnosis.

## 1. Introduction

The detection of nucleic acids (NAs) as specific markers of infectious diseases is commonly implemented in molecular biology laboratories which include sophisticated equipment and specialized professionals. The translation of these benchtop assays to a portable format, to be used by untrained personnel out of the laboratory, demands huge efforts of integration and automation. The last coronavirus pandemic has remarkably accelerated this R&D field and noteworthy solutions have emerged [1,2,3]. Nowadays, even though some remarkable developments have reached the market [4,5], there are still several technical issues that hinder further deployments.

Here we focus on a problem often driven by the modern molecular diagnosis that combines isothermal amplification, such as LAMP (loop-mediated isothermal amplification) or RPA (recombinase polymerase amplification) [6,7,8], and CRISPR (clustered regularly interspaced short palindromic repeats) based detection [9,10,11]. The integration of both reactions in microfluidic chips poses several challenges, whereas some limiting requirements usually need to be addressed: (i) After amplification, only a small fraction of the reaction products must be used for the subsequent reaction, typically an aliquot of 2–8 microliters [12,13,14,15,16]; (ii) the chambers containing amplificated NA strands cannot be opened to the ambient during the entire assay; (iii) the system must be safely sealed to prevent loss of material during the heating steps, typically 30 min at 64 °C for LAMP. Achieving (i) on microfluidic chips while satisfying the requirements (ii) and (iii) has not been reported yet in the open literature. To fulfill this technical problem, the present work proposed a novel aliquoting method for molecular diagnosis on chips.

The existing microfluidic approaches that integrate aliquoting on chips are briefly revised as follows. The first field to be mentioned is capillary electrophoresis, where the injection system is determined by the volume of the sample plug that is to be delivered to the separation process [17,18,19]. In these chips, the aliquot is defined by the geometric configuration of the microchannels (single-cross, double cross, double-L) and the timing of the electrokinetic pumping. Precisely, valving and external actuation are critical for metering. The second field came along with the possibility to fabricate micromechanical valves and pneumatic pumps from silicone elastomers, which led to robust and scalable microfluidic metering methods [20,21]. These chips strongly demand software and hardware, for programming and external control, respectively. Combinations of the above approaches (the metering strategy of electrophoresis on chips and pneumatic actuation) are also under study [22]. Nevertheless, the field that mastered aliquoting is centrifugal microfluidics [23], where volume metering is combined into a series of unitary operations to resolve complex assay programs [24], including the handling of multiple fluids [25]. Of course, these systems rely on CD-like chips and bulky equipment for the administration of controlled centrifugal forces. Finally, it is worth mentioning that, as far as valving is included, any reduction of external hardware becomes an extra task for the user, such as in the case of the finger-actuated devices that were reported for metering and dispensing [26,27,28].

Therefore, considering that system complexity and operation are relevant issues in the development of microfluidics cartridges for molecular diagnosis, we have designed an innovative operation to meter and aliquot in the microliter range, during a stepped assay, without requiring integrated valves. The operation is achieved by integrating a calibrated piece of porous material, which retains the desired amount of liquid after removal of the excess of reaction products. Microfluidic devices were prototyped and experimentally tested to demonstrate the overall performance (general fluidics, metering, aliquoting, mixing and reaction). It is worth noting that LAMP is perfectly achievable on porous materials, as demonstrated in several paper-based microfluidic devices [6,29], and the implementation of CRISPR/Cas detection on paper substrates is also currently being reported [30,31]. Furthermore, the integrated porous material could incorporate additional advantages, such as sample concentration, storage of dry reagents and material functionalization. Beyond NA assays, the proposed aliquoting method could be employed in different stepped microfluidic processes.

The paper is organized as follows. Section 2 presents the concept and some theoretical considerations. Section 3 describes the materials, experimental runs and data treatment. Then, Section 4 reports the results and discusses the main findings. Finally, Section 5 outlines some concluding remarks.

## 2. Theory

### 2.1. Concept and Design

**Figure 1:**
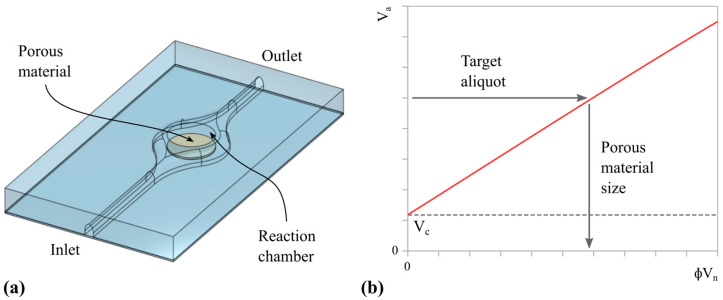
(**a**) Scheme of the microfluidic chip designed to implement valveless aliquoting and the necessary parts. (**b**) Plot of the effectively retained aliquot as a function of the nominal volume of the porous material, according to the theoretical correlation given by Equation (2).

The proposal is based on three main hypotheses. The first one is that an aliquot of liquid can be retained in a piece of porous material, after the excess liquid is removed by any pumping procedure normally used in microfluidics. It is well understood that porous materials autonomously capture liquids due to the high capillary pressures generated in the pores. Consistently, even larger pressures must be applied to drain the liquid from the porous material. In the case of Whatman #1 filter paper, drainage experiments made by centrifugation show that pressure drops (∆*P*) higher than 30 kPa are required to decrease water saturation to 80%, and higher than 100 kPa to reach 50% [32,33]. To prove this concept, the simplest design involves a microfluidic chamber enclosing a piece of porous material, connected to inlet and outlet ports for fluid manipulation, as shown in Figure 1a. This chip includes only the essential components for aliquoting; nevertheless, the operation can be modularly integrated to additional operations on chips or cartridges with more complex architectures.

The second hypothesis is that the retained amount of liquid (aliquot) can be controlled by the volume and the porosity of the porous material. For example, employing a paper disk with diameter *D* and thickness *h*, the retained volume will be *V*_r_ = *ϕV*_n_ + V_s_. The first term represents the fluid in the pore space, where *V*_n_ = *hπD*^2^⁄4 is the nominal volume of the disk and *ϕ* is the material porosity. The second term represents the fluid adsorbed on the disk surface. This phenomenon, also known as hemi-wicking [34], occurs when a liquid spreads on a surface because of the capillary forces associated to the surface rugosity, as well as to the presence of pillars, filaments or fibers. The adsorbed liquid volume is proportional to the disk exposed surface; thus, *V*_s_ = *dπD*^2^/4 + *dπDh*, where *d* is the effective film thickness. Note that the second term can be neglected if *h*/*D* << 1, which is the case for millimetric disks of Whatman #1 paper (*h* ≈ 180 μm). Therefore, the retained volume can be written as

*V*_r_ = *V*_n_ (*ϕ* + *δ*), (1)

where *δ* = *d*/*h* is a fraction to be determined experimentally. It is worth noting that, although these expressions were derived for disk-shaped pieces of paper, the concept is general and can be used to design chips with alternative porous materials and arbitrary geometries (note that Equation (1) is written in terms of volumes and dimensional fractions). For example, if a square prism (side *s*, height *h*) of nitrocellulose (*ϕ* ≈ 0.9) were used instead of a paper disk, then *V*_n_ = *hs*^2^ and *V*_s_ = *ds*^2^ + *d*4*sh*. Again, if the area of the square is sufficiently large (*h*/*s* << 1), the volume of water adsorbed at the prism perimeter can be neglected and *V*_s_ ≈ *ds*^2^. Thus, adding the partial contributions to compose *V*_r_ yields Equation (1), though *ϕV*_n_ and *V*_s_ come from a different geometry. In any case, it is worth highlighting the well-defined correlation between *V*_r_ and *D*^2^ or *s*^2^, which enables fluid volume metering by controlling the disk diameter or the square side length, respectively, for a given paper thickness.

The third hypothesis is that when a second liquid is injected into the chamber, it will progressively mix with the aliquot, firstly by advection and then by interdiffusion, and chemical reactions can rapidly take place. One should further note that the time for a small molecule to diffuse 180 μm (the scale of paper thickness) is of few seconds. In practice, mixing can be improved by applying external energy fields. Here, we experimentally demonstrate (see Section 4.2) that passive mixing and subsequent reaction is achieved in relatively short times.

### 2.2. The Influence of Corner Films

Microfluidic chips made by conventional microfabrication techniques frequently involve microchannels with trapezoidal cross-sections, with inner edges along the channels. Consequently, when the fluid inside a microchannel is replaced by another, namely air in our application, there is a certain amount of the former fluid that persists along the corners, hereafter denominated “corner films”. The effect is due to the strong capillary forces developed at the inner edges, mainly in acute angles [35]. The phenomenon also takes place in microfluidic chambers and reservoirs. The capillary pressure at the corners can be estimated by Laplace’s equation, ∆*P*_c_ ≈ 2*σ*⁄*r*_c_, where *σ* is the fluid surface tension and *r*_c_ is the curvature radius of the meniscus. For water, for example, a meniscus with curvature radius about 100 μm entails 2.7 kPa. This value sets an order of magnitude of the pressures required to evacuate the corner films.

Therefore, in the aliquoting method here proposed, after removing the main excess fluid, certain residual fluid volume is expected to remain in the corners (*V*_c_), apart from the volume retained in the porous material (*V*_r_). Finally, using Equation (1), the total retained aliquot (*V*_a_) results:

*V*_a_ = *V*_c_ + *ϕV*_n_ (1 + *δ*/*ϕ*). (2)

This function, the expected aliquot (*V*_a_) vs. the nominal retaining volume (*ϕV*_n_), is illustrated in Figure 1b, where one realizes the possibilities to design a well-defined fluid metering system. For better precision, an experimental calibration is required for both the intercept is (*V*_c_) and the curve slope (1 + *δ*/*ϕ*), as described below in Section 3.2.

### 2.3. The Operation Mechanisms

In order to remove the excess fluid from the reaction chamber, either a positive or negative pressure drop ∆*P* must be applied between the chip ports. The analysis above indicates that there are two characteristic pressure values to consider for the actuation of the aliquoting system: the maximum pressure to avoid draining the porous material (~30 kPa for water in Whatman #1 filter paper) and the typical Laplace pressure of the corner films, which is inversely proportional to the meniscus curvature. Therefore, the higher the applied ∆*P*, the lower the corner films volume, *V*_c_, provided the upper threshold is not reached.

Two operation mechanisms to remove the excess fluid are considered, depending on the driving force: flow rate-controlled and pressure-controlled fluid removal. In either case, the Poiseuille relation holds [36],

∆*P* = *Q*
*R*, (3)

where *Q* is the volumetric flow rate and *R* is the hydrodynamic resistance of the system. When *Q* is imposed, the resulting ∆*P* depends on *R* and, conversely, when ∆*P* is imposed, *R* determines the resulting *Q*. For the sake of simplicity, in our tests we made use of syringes actuated by hand, but the aliquoting operation can be readily coupled to any automated pumping system. Nevertheless, it is worth noting that the operation mechanism influences the attained aliquot. In the *Q*-controlled fluid removal, for example, if the syringe plunger is pulled at the speed that produces *Q* = 1 mL/s, for *R* = 10^9^ Pa·s·m^−3^, the maximum pressure drop is 1 kPa, according to Equation (3). This ∆*P* will remove the bulk fluid, but will not fully evacuate the corner films. In the ∆*P*-controlled fluid removal, a standard syringe with a Luer stopcock enables the generation of vacuum pressures about 10–20 kPa [37]. These values are still below the threshold to avoid paper drainage, but are large enough to drag fluid from the corner, and hence to reduce the final *V*_c_ (intercept in Figure 1a).

## 3. Materials and Methods

### 3.1. Fabrication of Microfluidic Chips

**Figure 2:**
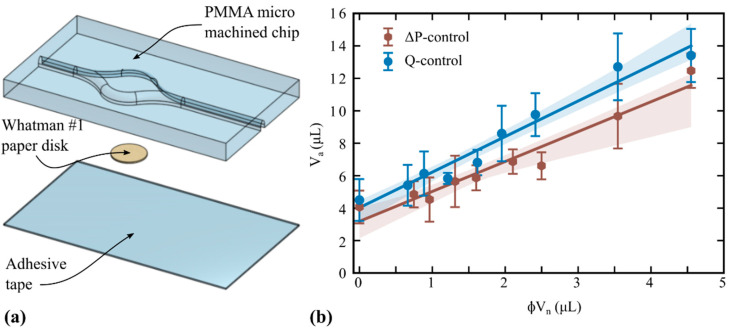
(**a**) Exploded view of the microfluidic chip incorporating a cut paper disk for aliquoting. (**b**) Retained aliquot as a function of the nominal retained volume (different disk diameters), for both liquid extraction mechanisms. Solid circles are experimental data (average values), the bars being the standard deviation (*n* = 10). Solid lines are the prediction of Equation (2) with the parameters reported in Table 1. The shaded bands represent the fitting 95% confidence interval.

The main body of the microfluidic chips (Figure 1a) was fabricated on a 1.8 mm thick PMMA sheet. PMMA engraving and cutting were made by CO_2_ laser micromachining (Lasers Cuyana, San Rafael, Argentina). The chamber diameter was 8 mm. The chamber and channels height were 1.5 mm, while the channels width and length were 1 and 15 mm, respectively. All the edges were rounded to decrease liquid retention. The chosen porous material was Whatman #1 filter paper (Cytiva, Marlborough, MA, USA). In order to demonstrate the concept for different configurations, as well as to provide alternatives for the integration of the porous material into the polymeric device, two different strategies were studied: the first one makes use a paper disk attached to the bottom surface of the chamber, while the second one makes use of wax-printed paper to constitute the bottom surface and delimitate the porous disk in the chamber. For the first strategy, paper disks were cut into different diameters ranging from 2 to 8 mm using a punching machine. Regular adhesive tape (AUCA 18 mm, FAMYCA, Villa Madero, Argentina) was used to seal the PMMA body and attach the disk to the chamber center (see Figure 2a). It is worth adding that commercial adhesive tapes specially designed to be compatible with molecular reactions are available, and could be used.

**Figure 3:**
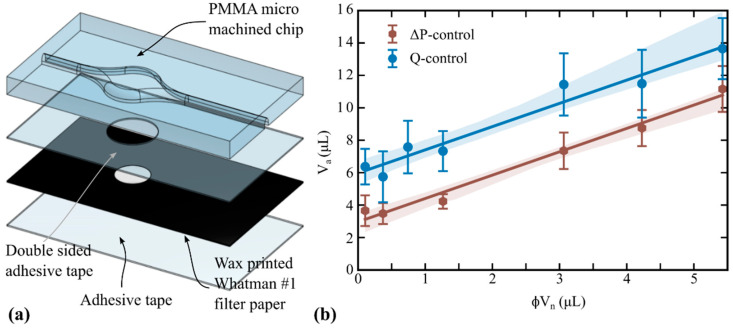
(**a**) Exploded view of the microfluidic chip incorporating a wax-printed paper disk for aliquoting. (**b**) Retained aliquot as a function of the nominal retained volume (different disk diameters), for both liquid extraction mechanisms. Solid circles are experimental data (average values), the bars being the standard deviation (*n* = 10). Solid lines are the prediction of Equation (2) with the parameters reported in Table 1. The shaded bands represent the fitting 95% confidence interval.

For the second strategy, paper sheets with the same area than the PMMA body were wax-printed (ColorQube 8580, Xerox, Norwalk, CT, USA), covering the full area except for a circle with diameters ranging from 2 to 8 mm, concentric with the chamber. After melting the wax in a hot plate (~60 s, 130 °C), the bottom side of the paper sheets was covered by adhesive tape. Then, the wax-printed paper layers and the PMMA body were aligned and glued together by using double-sided adhesive tape, which had been perforated concentric to the chamber (see Figure 3a). Bonding by adhesive tape was implemented in order to re-use the devices after replacing the retaining material, which is a practical solution for the purposes of this experimental program. Nevertheless, other bonding options are compatible with the proposed aliquoting method, provided a porous material is suitably integrated in the chamber.

### 3.2. Calibration of Volume Metering

For a given microfluidic chip and porous material, the aliquoting methodology requires a precise calibration step to determine the retained fluid volume for a given nominal volume of the paper disk. For this purpose, the conventional method of mass difference was implemented by using an analytical balance (Boeco BAS31, Hamburg, Germany). Briefly, the mass of the dry microfluidic chip was initially determined. Then, the chamber was filled with DI water, the excess liquid was removed (more details on this step are given below) and the mass of the chip with the imbibed porous material was determined. Finally, the mass difference (before-after imbibition) was converted to the liquid volume (aliquot), taking into account the water density at the corresponding temperature. All the experiments were conducted in a laboratory with controlled conditions: room temperature was set at 24 °C and relative humidity was about 40–50%. Regarding the removal of excess liquid after imbibition, both mechanisms described in Section 2.2 were used.

For the *Q*-controlled fluid removal, a 1 mL syringe was connected to the chip through a 21G cannula. This capillary tube becomes the controlling hydrodynamic resistance of the system, with *R* ≈ 10^9^ Pa.s.m^−3^ for water and around 10^7^ Pa·s·m^−3^ for air at room temperature. The syringe plunger was pulled at a relatively uniform speed, which corresponds to a given *Q*. After the fluid had been removed, air was flowed through the chamber by the same syringe; nevertheless, the resulting pressure drop was still lower for air and the corner films were stable, even at relatively large flow rates.

For the ∆*P*-controlled fluid removal, a Luer stopcock was coupled to a 5 mL syringe, then the plunger was pulled (with the stopcock closed) to create a 2 mL void volume into the syringe. To start fluid extraction, the stopcock was suddenly opened and the system was allowed to evolve autonomously, until the syringe reached atmospheric pressure. For standard plastic syringes, a plunger displacement of 2 mL generates a vacuum pressure of 25 kPa, approximately [37].

Experiments were made with the two chip alternatives (cut and wax-printed paper disks), applying the two different fluid handling mechanisms (*Q* or ∆*P*-controlled removal), for a series of disk sizes, and performing ten repetitions for each measurement, making around 300 data points.

### 3.3. Visualization of Mixing and Reaction

The feasibility of mixing and reaction from the retained aliquot was tested as follows. A dilute NaOH solution (pH = 10) was loaded into the chamber and then removed to retain a ~10 μL aliquot. Subsequently, a phenolphthalein (Phph) solution (pH = 7) was loaded into the chamber. Phph undergoes a color shift, from transparent to pink, when the solution pH reaches 8 and above. Thus, the onset of pink color in the chamber can be used as an indicator of effective mixing between the first and second reagent, as demonstrated in previous analysis on acid-base titrations in wax-printed paper [38]. Images at different assay times were captured with a smartphone Xiaomi Redmi Note 8 equipped with a high-resolution camera. The experiments were made with both alternative chips (cut and wax-printed paper) operated under *Q*-controlled conditions.

## 4. Results and Discussion

### 4.1. Aliquoting

In order to prove the proposed aliquoting method, the different combinations of alternative chips (cut or wax-printed paper disks) and liquid removal methods (*Q* or ∆*P*-controlled) were tested. The results obtained with cut paper disks are reported in Figure 2b, where the measured aliquot (*V*_a_) is plotted against the nominal retained volume (*ϕV*_n_). Note that each data point is the mean value of ten measurements, with the error bars representing the standard deviation. In the same figure, the lines are the regression curves according to Equation (2), with the parameter values reported in Table 1: the aliquot-axis intercept is *V*_c_ (μL) and the curve slope is 1 + *δ*/*ϕ* (dimensionless). It should be remarked that measurements confirm the linear trend between *V*_n_ and the effectively retained aliquot, as predicted by Equation (2). Furthermore, the high coefficient of determination (Table 1) ensures that the proposed design can be safely calibrated to meter pre-defined aliquots.

According Equation (2), the curve slope should be 1 in the absence of hemi-wicking (*δ* = 0). In Figure 2b, the resulting curve slope was around 2 (Table 1), hence *d*/(*ϕh*) ≈ 1, meaning that the amount of liquid captured by hemi-wicking is similar to that retained inside the disk. It is also interesting that the curve slope results were slightly lower for the ∆*P*-controlled liquid removal, suggesting that this mechanism extracted part of the hemi-wicking component. Regarding the aliquot-axis intercept, one notes that the corner films are also reduced by ∆*P*-controlled removal, as expected, following the reasonings in Section 2.3.

**Table 1:** Parameters of Equation (2) for the calibration experiments (Figure 2b and Figure 3b) and the respective coefficient of determination r^2^.

Chip Type	Operation	(1 + *δ*/*ϕ*)	*V*_c_ [μL]	r^2^
Cut paper (Figure 2)	*Q*	2.19	4.03	0.97
	∆*P*	1.84	3.19	0.98
Wax-printed paper (Figure 3)	*Q*	1.43	5.97	0.97
	∆*P*	1.44	2.97	0.99

The results obtained with wax-printed paper disks and both actuation mechanisms are reported in Figure 3b. In this retention strategy, the curve slopes resulted (i) notably lower than those in Figure 2b and (ii) invariant with the fluid removal mechanism (see Table 1). Both outcomes indicate that the contribution of hemi-wicking is lower than that in the cut paper format, which is reasonable if one considers that, in the wax printed format, the retention disks are in the surface of the chamber floor (not over the surface) and surrounded by a hydrophobic boundary.

Regarding the aliquot-axis intercept, it was found that the chip assembled with wax-printer paper and double-sided tape involved a larger *V*_c_ (Table 1); however, it was also found that these corner films, mainly present at the intersection of the PMMA body and the adhesive tape, were further reduced by ∆*P*-controlled removal (Figure 3b), in analogy to the case of cut paper disks (Figure 2b). To further decrease *V*_c_, two practical ways could be followed: rounding the remaining chip edges from microfabrication and/or increasing the applied pressure during the liquid removal step. The former case is ultimately limited by the rugosity of the chip material. The latter case is limited by the drainage pressure of the porous material (the upper pressure threshold).

The plots in Figure 2b and Figure 3b comprise the calibration curves for the four chip/actuation combinations. In any case, the aliquot-axis intercept of the regression curve represents the liquid retained in the chip, apart from that retained in the porous media. Furthermore, *V*_c_ was independently measured as the amount of retained liquid when no disk was included in the chamber; the values are those plotted at *ϕV*_n_ = 0 in Figure 2b. It is worth remarking that the measured values coincide fairly with the curve intercept, showing the consistency of the method with the theoretical model. In both plots, *V*_c_ resulted lower for the ∆*P*-controlled than the *Q*-controlled liquid removal, meaning that the applied pressure was larger and more efficient to drag fluid along the inner edges, as predicted in Section 2.2. Also, the precision of the aliquoting was found to be better with the ∆*P*-controlled liquid removal, according to the relative size of the error bars. Concerning the curve slopes in Figure 2b and Figure 3b, one may observe that they depend on the geometric configuration of the porous material (cut or wax-printed disks) rather than on the liquid removal mechanism.

In this work, we have proved the aliquoting method by employing paper disks, but in principle there are no technical restrictions to the shape of the retaining porous material, nor to the material itself, provided it is affine to the working fluid. In any case, it is important to highlight that, once the whole system is defined (chip geometry, chip actuation, porous material, working fluid, assay parameters), a calibration procedure is mandatory, in order to account for the potential effect of hidden factors, such as fluid characteristics (surface tension, viscosity) or in the case of extreme assay conditions (temperature, pressure).

### 4.2. Mixing and Reaction

**Figure 4:**
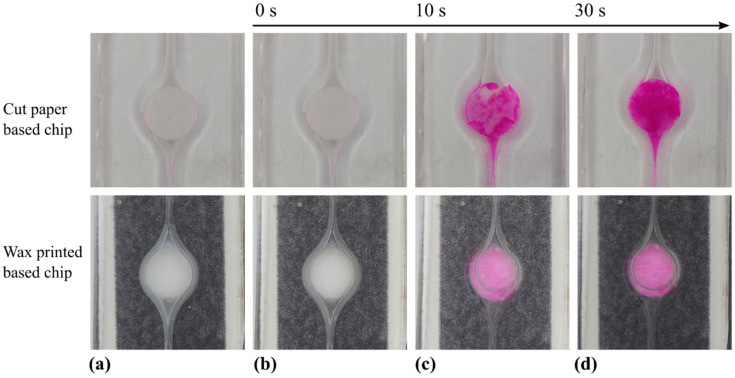
Images of stepped operations for aliquoting, mixing and reaction. The image sequences in the upper and lower lines correspond to devices with cut and wax-printed paper, respectively. In both cases: (**a**) Aliquoting about 10 μL of a NaOH solution at pH = 10. (**b**) Filling the chamber with a Phph solution at pH = 7. (**c**) Color development at 10 s. (**d**) Color development at 30 s.

Figure 4 presents the results from the experiments that demonstrate the achievement the sequential operations of aliquoting, mixing and reaction. Briefly, an aliquot of alkaline solution (pH 10) was retained in the disk (Figure 4a) and then the chamber was filled with a transparent Phph solution (Figure 4b). The solution shifted from transparent to pink (Figure 4c), meaning that Phph was mixed enough to get pH > 8. The color visibly developed in less than 10 s and gradually progressed to become uniform in the full chamber (Figure 4d). It is worth remarking that the color only arises after the acid–base reaction occurs, indicating that effective mixing between the first and second reagent took place [38].

The experiments reported in Figure 4 show how the proposed aliquoting method enables passive mixing and chemical reaction between the aliquoted reagent and another reagent injected to the chamber. This simple example demonstrates the feasibility of implementing sequential molecular reactions, or any other type of chemical reactions, requiring aliquoting and sequential steps. The integration of aliquoting into a whole set of operations is further illustrated next.

### 4.3. Aliquoting in Molecular Diagnosis

As a suitable application example, here we consider the on-chip detection of NA by LAMP and CRISPR/Cas, which was introduced in Section 1. The posed problem was that only a small fraction of the amplification products (typically 2–8 μL) must be used for the subsequent reaction [12,13,14,15,16]. It was also anticipated in Section 1 that the present work was focused on proposing a microfluidic solution for this technical problem.

**Figure 5:**
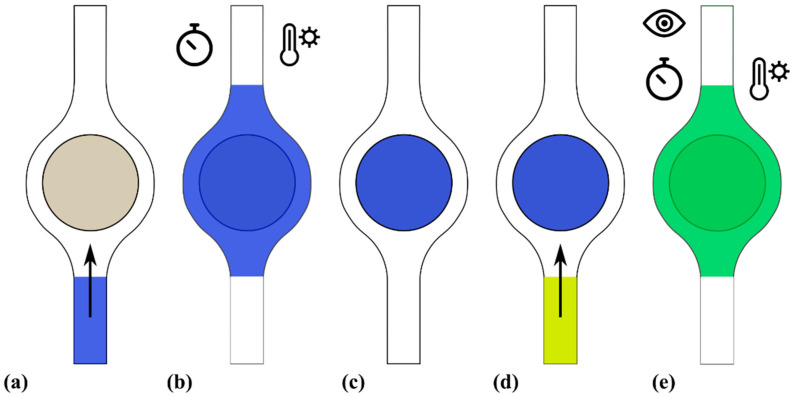
Schematic representation of a series of operations that integrates aliquoting: (**a**) Injection of the first reagent (blue) into the chamber. (**b**) First reaction during a given time at a specified temperature. (**c**) Removal of the excess liquid and aliquoting in the porous material. (**d**) Injection of the second reagent (yellow) into the chamber. (**e**) Mixing (green), second reaction during a given time at a specified temperature, and final readout.

The steps proposed to implement aliquoting and sequential reactions are schematically shown in Figure 5. Initially, the first liquid is loaded to the completely dry reaction chamber (Figure 5a). Such liquid is a mix of the sample (target) and LAMP reagents. Then, the amplification reaction is carried out (Figure 5b), requiring 20–60 min at 60–68 °C. After that, the reaction product is removed from the chamber, leaving the aliquot retained in the porous material (Figure 5c). Depending on the design of the full system, the excess liquid can be removed in any direction, either to a waste reservoir or to be used elsewhere. Next, the CRISPR/Cas reagents are introduced into the chamber (Figure 5d). The reagents mix with the reaction products retained in the porous material (Figure 5e) and the detection reaction is carried out, requiring about 10 min at 37 °C. The assay ends once an optical signal develops (color shift or fluorescence), which can be read directly from the chamber.

Finally, it is worth remarking that the whole process demands minimum operation and can be achieved without valves. As mentioned in Section 1, both LAMP [6,29] and CRISPR/Cas [30,31] are perfectly compatible with paper-based microdevices. In addition, the device can be easily adapted to satisfy the additional requirements of NA tests: keeping the reaction chamber closed during the entire assay and sealing the system to prevent material loss during incubation at high temperatures.

## 5. Conclusions

In summary, here we have reported the concept, design, prototyping and assessment of a microfluidic device that is able to meter and aliquot in the microliter range during sequential assays. The aliquoting operation is achieved by integrating a porous material that retains the desired amount of liquid, avoiding valves and external actuation. The proposed aliquoting method is compatible with the sequential reactions required for NA analysis, mainly for CRISPR/Cas-based determinations preceded by LAMP or RPA amplifications. Moreover, this methodology can be further exploited by including additional features like functionalizing the porous material, using the pore space to store dry reagents, or to concentrate NA.

From the conceptual point of view, it is worth remarking that we have proved the three main hypotheses made for the working principle of the aliquoting method (Section 2.1). The confirmation of these hypotheses was described throughout Section 4.1 and Section 4.2: Figure 2 and Figure 3 show that aliquots were retained in the paper disks after excess liquid removal and that the aliquot can be quantitatively predicted; Figure 4 shows how an aliquot of a given fluid mixes and reacts with a second reagent. Accordingly, the theoretical bases of the device operations were discussed throughout the work, providing a solid background for further exploration of the method, as well as for adaptations to alternative on-chip applications with specific requirements for retention or liquid removal.

From the fabrication point of view, one should note that several innovations could be made. For example, the prototypes here tested were able to meter fluid volumes in the range of 3–14 μL; however, this range can be expanded by modifying the shape and size of the porous material, as well as the material itself. In this regard, an interesting possibility is taking advantage of the liquid retention capability of the chip inner edges, disregarding the extra porous material. Furthermore, the nowadays technologies for micropatterning (micro wells or micro pillars) and 3D printing (powders and porous structures) could be explored to integrate designed liquid retention sections, directly micromachined on the chip body, and then calibrated to obtain the desired aliquot.

## 6. Patents

A provisional patent application was filed in Argentina to protect the intellectual property of this work. Provisional patent application number: 20210102760.

## Data Availability

The data presented in this study are available on reasonable request from the corresponding author.
